# Supplementary material for: SNRK3.15 Is a Crucial Component of the Sulfur Deprivation Response in Arabidopsis thaliana
Source: Plant Direct. 2025 Dec 28;10(1):e70132. doi: 10.1002/pld3.70132 (PMC12744938; doi:10.1002/pld3.70132)
Supplement: Supplementary file 2 — Figure S1: Supplemental characterization of snrk3.15 lines, SNRK3.15 expression, and comparative growth in soil. Figure S2: Phenotypes of dry seed from Col‐0 and snrk3.15 plants grown on soil. Figure S3: Response of chlorophyll and chlorophyll degradation genes to –S in Col‐0 and snrk3.15 seedlings. Figure S4: Levels of proteins positively associated with chlorophyll content. Figure S5: Levels of proteins negatively associated with chlorophyll content. Figure S6: Exploratory analysis of global proteome. Figure S7: Levels of –S responsive, snrk3.15‐specific DAPs. Figure S8: Relationship between sulfate, OAS, and OAS‐cluster genes. [file PLD3-10-e70132-s001.pdf]

## Supplemental Figure Captions

### Supplemental Figure 1: Supplemental characterization of *snrk3.15* lines, *SNRK3.15* expression, and comparative growth in soil

(a) Diagram of the Arabidopsis *SNRK3.15* gene. Exon is indicated as a solid black box. The position of the T-DNA insertion is shown for both T-DNA mutant lines (SALK_09699 and SALK_147899), while the arrows represent the orientation of the insertion. (b) *SNRK3.15* transcript level relative to *TIP41* and *PP2A*, n = 5 biological replicates. Bar height and error bars correspond to the mean +/- SE. Within each treatment and timepoint, differences in means between Col-0 and each *snrk3.15* mutant were assessed by Welch two sample t-test. Asterisks indicate a significant difference (* $P < 0.05$; ** $P < 0.01$; *** $P < 0.001$). (c) Photo of representative Col-0 and *snrk3.15* mutant rosettes at 23 DAS grown in the greenhouse under long day conditions (16h day - 8h night). (d) Rosette area of Col-0 and *snrk3.15* mutants, grown under long day conditions (16h day - 8h night) in the greenhouse, n = 5 rosettes. Bar height and error bars correspond to the mean +/- SE. Within each timepoint, differences in means between Col-0 and each *snrk3.15* mutant were assessed by Welch two sample t-test. ns indicates no significant difference ($P > 0.05$).

### Supplemental Figure 2: Phenotypes of dry seed from Col-0 and *snrk3.15* plants grown on soil

Mature seeds were collected from siliques and dried in a drying room with humidity maintained at 15% and temperature at 15°C. (a) Mean single seed weight was calculated from the weight of 50 seeds, n = 5 independent plants. (b) Elemental profile of dry seeds, as determined by inductively coupled plasma mass spectrometry, n = 3 independent plants. Bar height and error bars correspond to the mean +/- SE. Differences among means were assessed by ANOVA followed by post hoc Tukey's pair-wise mean comparison testing with compact letter display, alpha = 0.05.

### Supplemental Figure 3: Response of chlorophyll and chlorophyll degradation genes to -S in Col-0 and *snrk3.15* seedlings

(a) Chlorophyll-a and (b) chlorophyll-b content in Col-0 and *snrk3.15* mutants, n = 3 biological replicates. Transcript level of chlorophyll degradation genes, (c) *NYC1* and (d) *SGR1* relative to *TIP41* and *PP2A*, n = 4-5 biological replicates. Within each treatment and timepoint, differences in means between Col-0 and each *snrk3.15* mutant were assessed by Welch two sample t-test. Asterisks indicate a significant difference (* $P < 0.05$; ** $P < 0.01$; *** $P < 0.001$).

**Supplemental Figure 4: Levels of proteins positively associated with chlorophyll content**

The normalized signal intensity of selected proteins in the proteomics dataset is shown. Each point corresponds to a biological replicate. Point shape corresponds to protein fraction, point fill corresponds to condition (3 DAT to -S or 3 DAT to FN), and point color corresponds to genotype. Proteins potentially positively associated with chlorophyll levels were selected based on their having been annotated to at least one of the following gene ontology terms: chlorophyll biosynthetic process (GO:0015995), magnesium chelatase complex (GO:0010007), magnesium chelatase activity (GO:0016851), positive regulation of chlorophyll biosynthetic process (GO:1902326), regulation of chlorophyll biosynthetic process (GO:0010380), protoporphyrinogen IX biosynthetic process (GO:0006782).

**Supplemental Figure 5: Levels of proteins negatively associated with chlorophyll content**

The normalized signal intensity of selected proteins in the proteomics dataset is shown. Each point corresponds to a biological replicate. Point shape corresponds to protein fraction, point fill corresponds to condition (3 DAT to -S or 3 DAT to FN), and point color corresponds to genotype. Proteins potentially negatively associated with chlorophyll levels were selected based on their annotation to at least one of the following gene ontology terms: chlorophyll catabolic process (GO:0015996), regulation of chlorophyll catabolic process (GO:0010271), negative regulation of chlorophyll biosynthetic process (GO:1902325).

**Supplemental Figure 6: Exploratory analysis of global proteome**

(a) PCA scores plot illustrating the distribution of samples in the first two principal component space (PC1 and PC2). The percent of total variance explained by each component is shown on the relevant axis in parentheses. Each point corresponds to a sample. Point shape represents protein fraction, point fill corresponds to condition (3 DAT to -S or 3 DAT to FN), and point color corresponds to genotype. PCA by singular value decomposition was performed on pareto scaled, imputed normalized intensity data matrix. (b) Histograms of the number of proteins detected in each sample. Samples from microsomal and soluble protein fractions are shown separately. Far fewer proteins were detected in the microsomal fraction sample *snrk3.15*_-S_rep2, as indicated. (c) Density plots of the normalized intensity values (log10 transformed) of the proteins quantified in at least 6 of 12 samples. Samples from microsomal and soluble protein fractions are shown separately. The sample *snrk3.15*_-S_rep2 has a density trace that is shifted relative to the other 11 microsomal fraction samples, as indicated.

**Supplemental Figure 7: Levels of -S responsive, snrk3.15-specific DAPs**

The normalized signal intensity of those proteins that were found to be differentially abundant in 3 DAT to -S compared to 3 DAT to FN in *snrk3.15.1* but not in Col-0 is shown. Only soluble fraction data are shown, because the 22 proteins that met the snrk3.15-specific and -S responsive criteria did so in the soluble fraction, but not the microsomal fraction samples. No proteins identified in the microsomal faction met the snrk3.15-specific and -S responsive criteria. Each point corresponds to a biological replicate. Point fill corresponds to condition, and point color corresponds to genotype.

**Supplemental Figure 8: Relationship between sulfate, OAS, and OAS-cluster genes**

Scatter plots of OAS concentration (a) or OAS-cluster gene expression (b-f) in relation to sulfate concentration. All data were log10 transformed prior to plotting. Each point corresponds to a single biological sample. Point color corresponds to the genotypic line of the sample. Point shape corresponds to the sample timepoint, days after transfer (DAT). Point fill corresponds to the condition after transfer.

## Figure S1

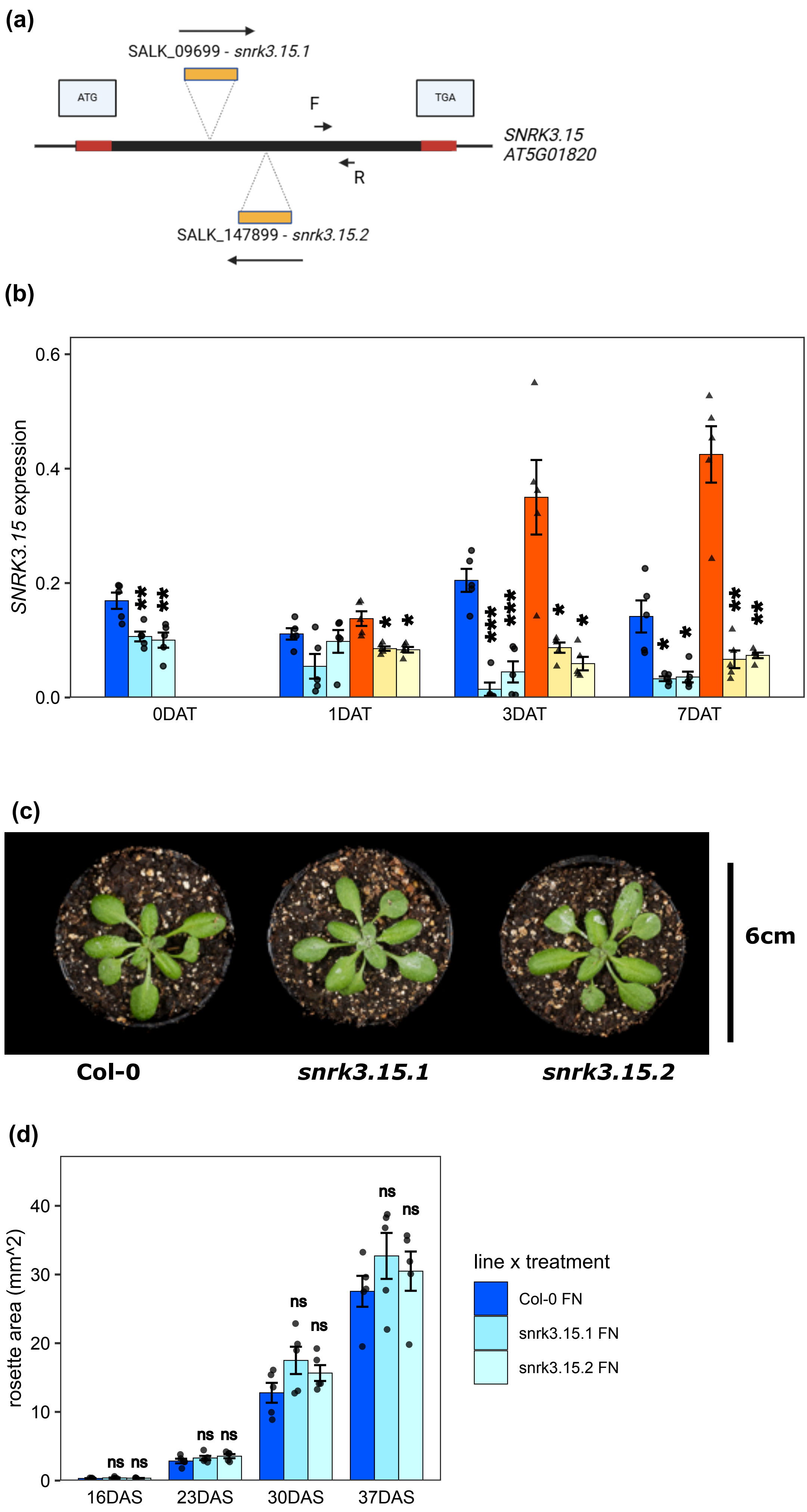

(a)
SALK_09699 - snrk3.15.1
ATG
TGA
F
R
SNRK3.15
AT5G01820
SALK_147899 - snrk3.15.2
(b)
SNRK3.15 expression
0.0
0.2
0.4
0.6
0DAT
1DAT
3DAT
7DAT
line x treatment
Col-0 FN
snrk3.15.1 FN
snrk3.15.2 FN
Col-0 -S
snrk3.15.1 -S
snrk3.15.2 -S
treatment
FN
-S
(c)
6cm
Col-0
snrk3.15.1
snrk3.15.2
(d)
rosette area (mm^2)
0
10
20
30
40
16DAS
23DAS
30DAS
37DAS
ns
line x treatment
Col-0 FN
snrk3.15.1 FN
snrk3.15.2 FN

# Figure S2

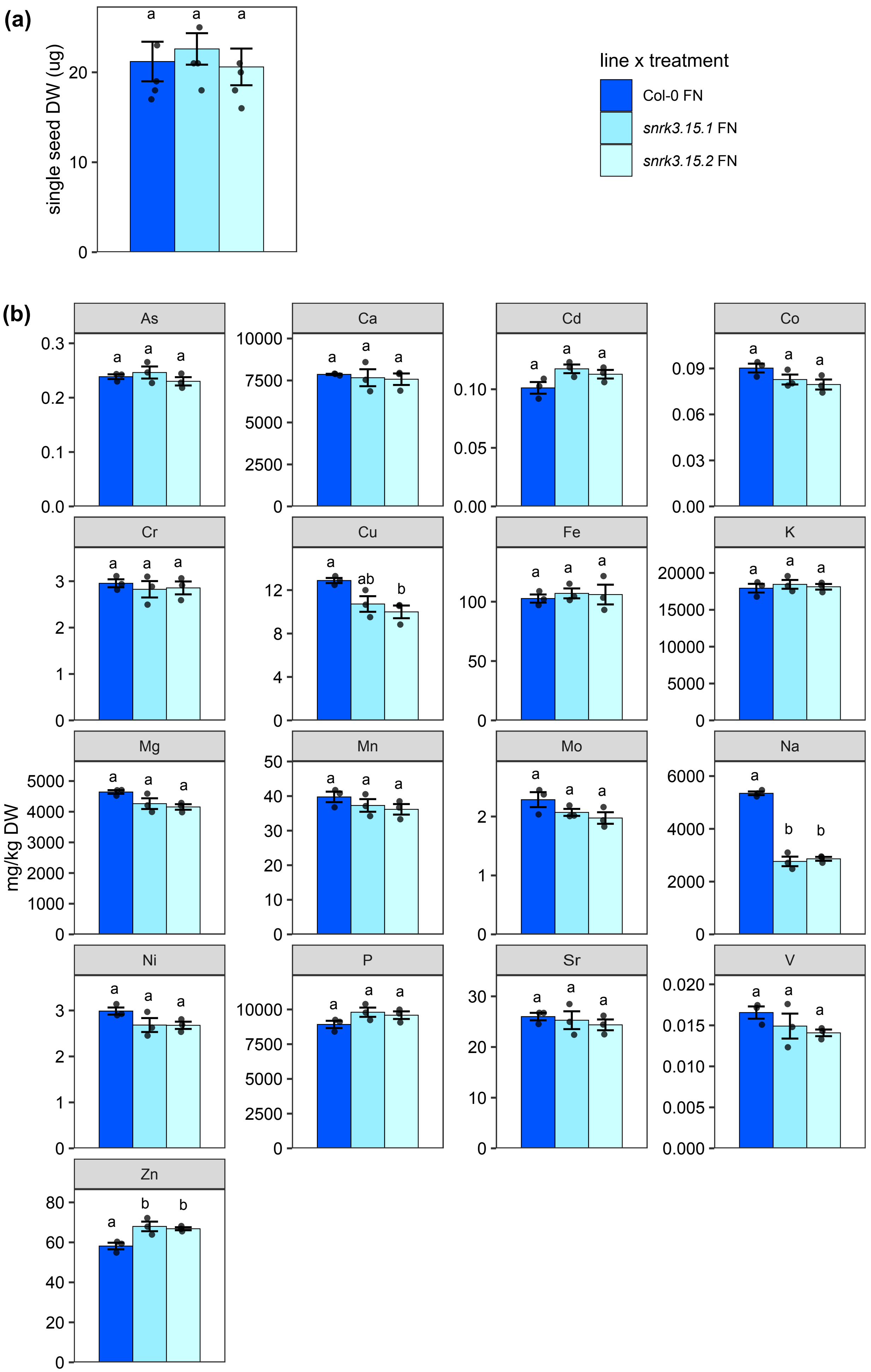
(a)
single seed DW (ug)
0
10
20
a
a
a
line x treatment
Col-0 FN
snrk3.15.1 FN
snrk3.15.2 FN
(b)
mg/kg DW
As
Ca
Cd
Co
Cr
Cu
Fe
K
Mg
Mn
Mo
Na
Ni
P
Sr
V
Zn
a
ab
b

# Figure S3

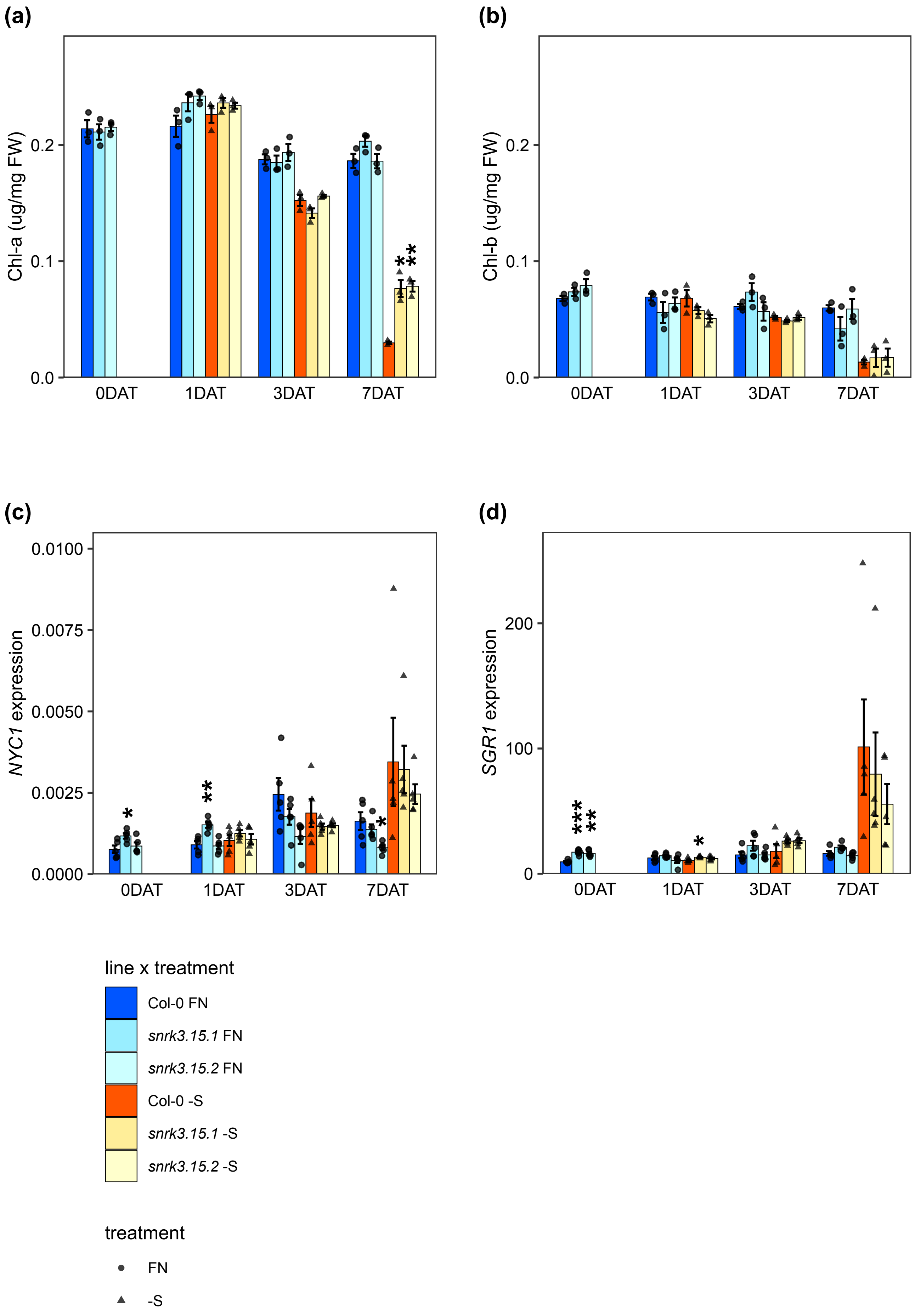

(a)
Chl-a (ug/mg FW)
0.0
0.1
0.2
0DAT
1DAT
3DAT
7DAT
(b)
Chl-b (ug/mg FW)
0.0
0.1
0.2
0DAT
1DAT
3DAT
7DAT
(c)
NYC1 expression
0.0000
0.0025
0.0050
0.0075
0.0100
0DAT
1DAT
3DAT
7DAT
(d)
SGR1 expression
0
100
200
0DAT
1DAT
3DAT
7DAT
line x treatment
Col-0 FN
snrk3.15.1 FN
snrk3.15.2 FN
Col-0 -S
snrk3.15.1 -S
snrk3.15.2 -S
treatment
FN
-S

**Figure S4**

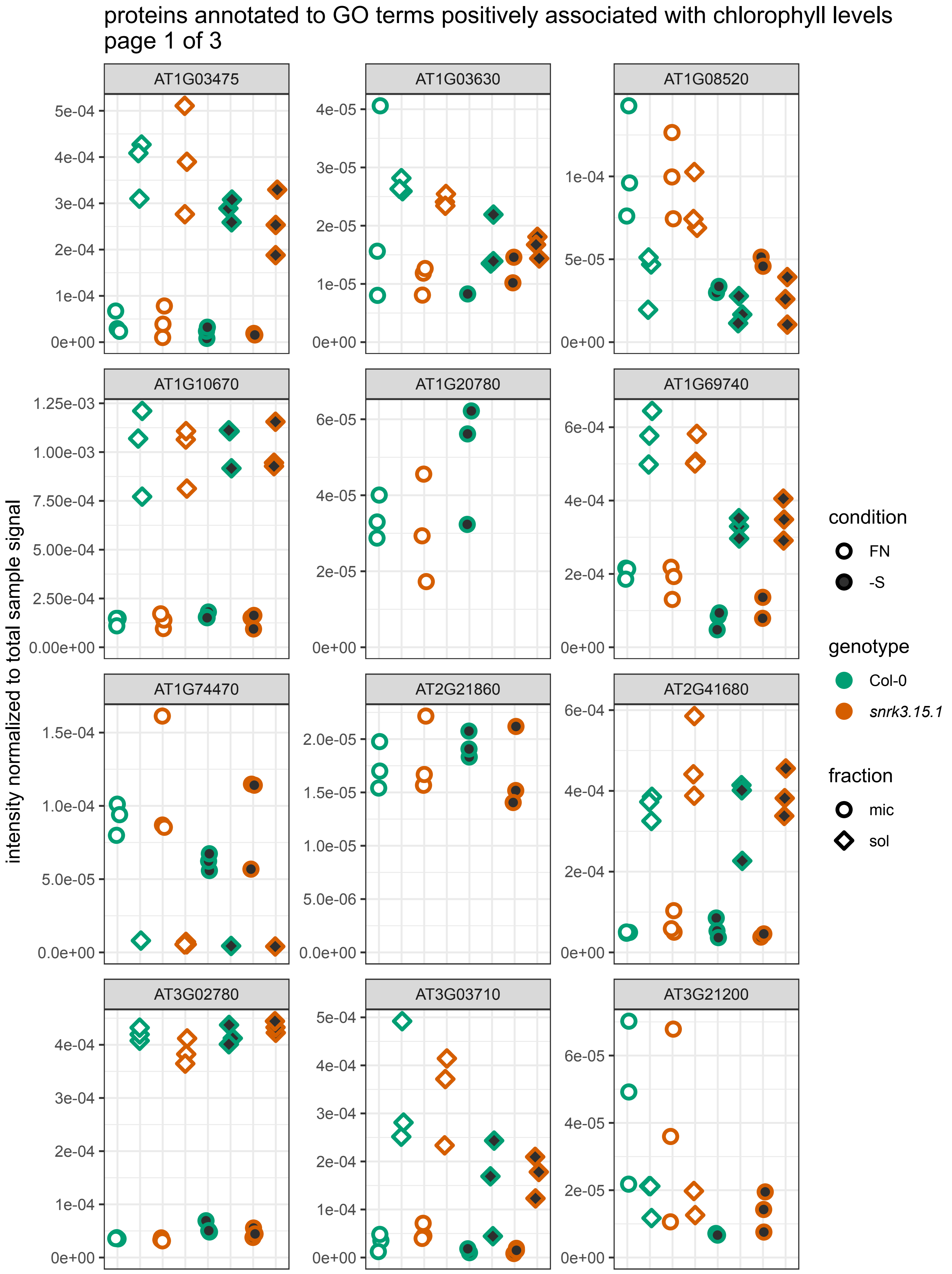
proteins annotated to GO terms positively associated with chlorophyll levels
page 1 of 3
AT1G03475
AT1G03630
AT1G08520
AT1G10670
AT1G20780
AT1G69740
AT1G74470
AT2G21860
AT2G41680
AT3G02780
AT3G03710
AT3G21200
intensity normalized to total sample signal
condition
FN
-S
genotype
Col-0
snrk3.15.1
fraction
mic
sol

proteins annotated to GO terms positively associated with chlorophyll levels
page 2 of 3

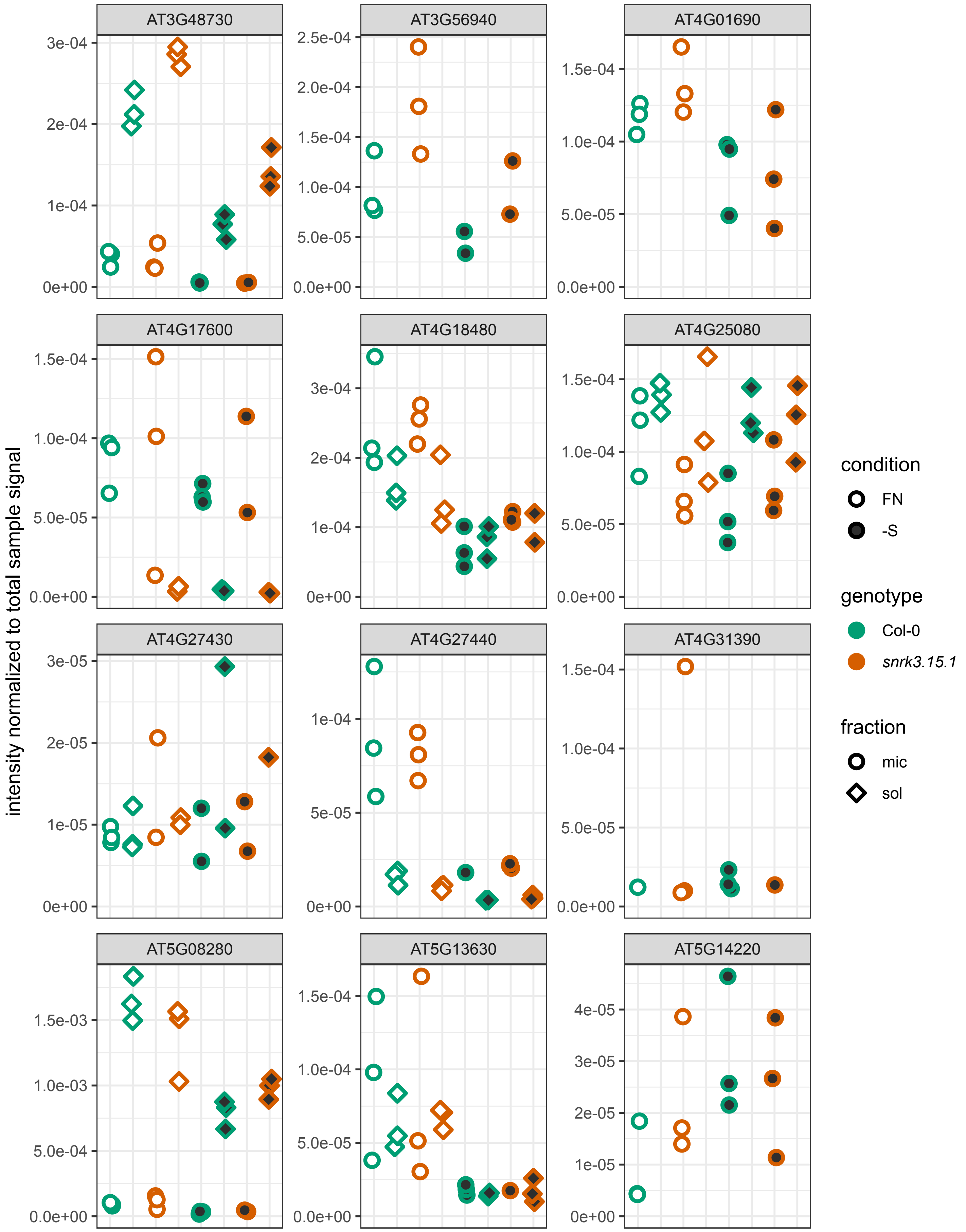

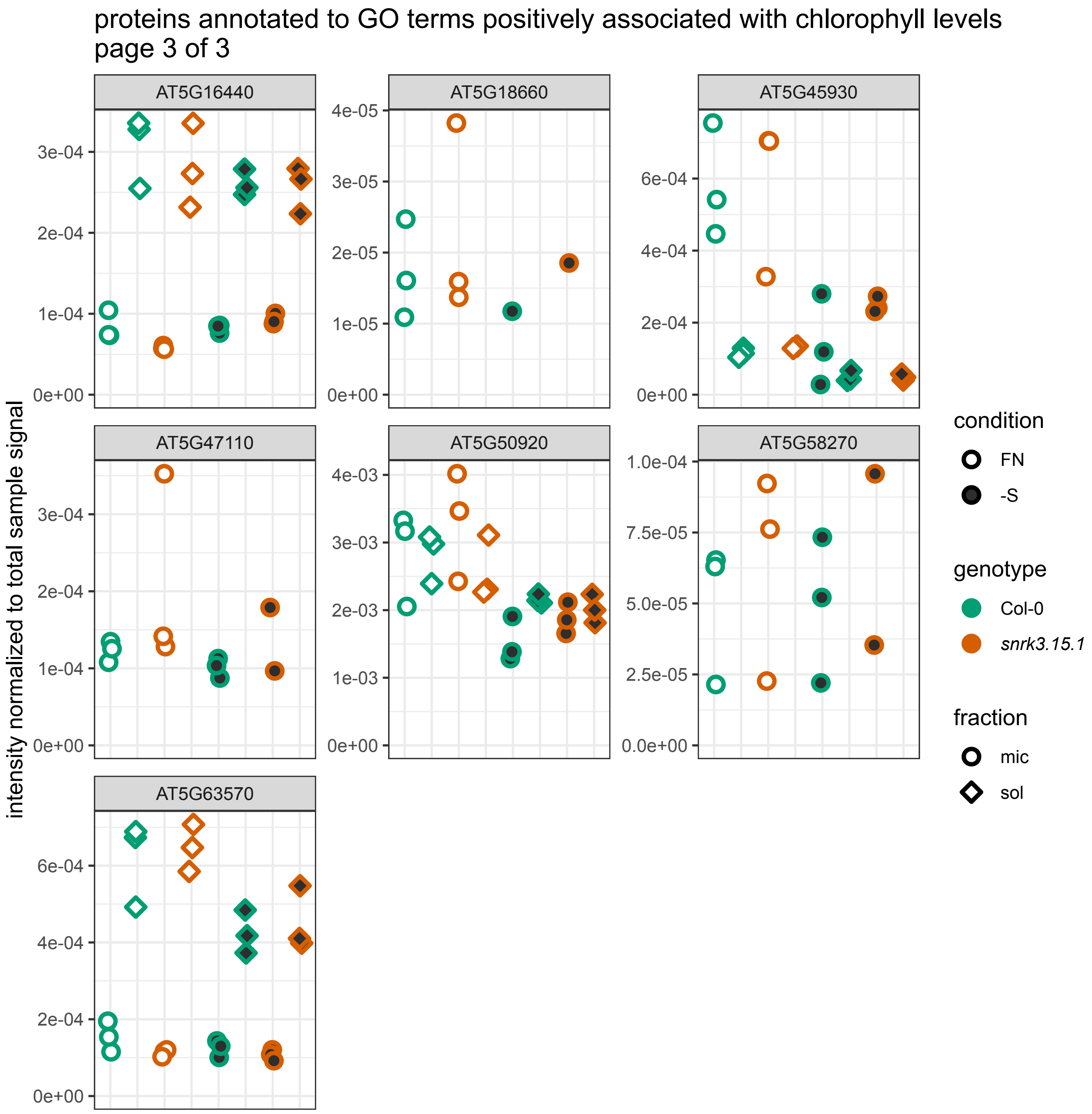

proteins annotated to GO terms positively associated with chlorophyll levels
page 3 of 3
AT5G16440
AT5G18660
AT5G45930
AT5G47110
AT5G50920
AT5G58270
AT5G63570
intensity normalized to total sample signal
condition
FN
-S
genotype
Col-0
snrk3.15.1
fraction
mic
sol

**Figure S5**

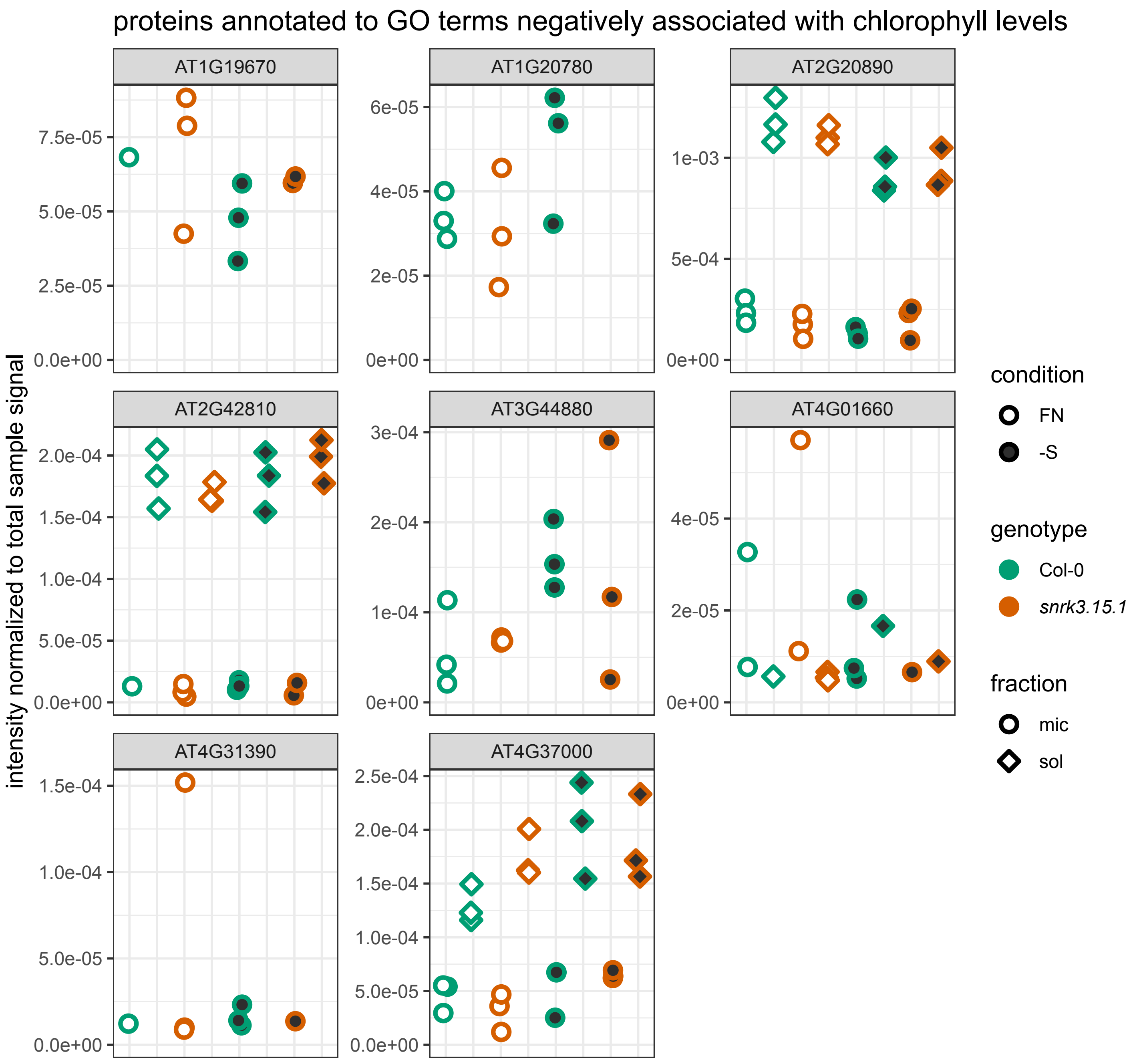

proteins annotated to GO terms negatively associated with chlorophyll levels
AT1G19670
AT1G20780
AT2G20890
AT2G42810
AT3G44880
AT4G01660
AT4G31390
AT4G37000
intensity normalized to total sample signal
7.5e-05
5.0e-05
2.5e-05
0.0e+00
6e-05
4e-05
2e-05
0e+00
1e-03
5e-04
2.0e-04
1.5e-04
1.0e-04
5.0e-05
3e-04
2e-04
1e-04
4e-05
2e-05
1.5e-04
1.0e-04
2.5e-04
condition
FN
-S
genotype
Col-0
snrk3.15.1
fraction
mic
sol

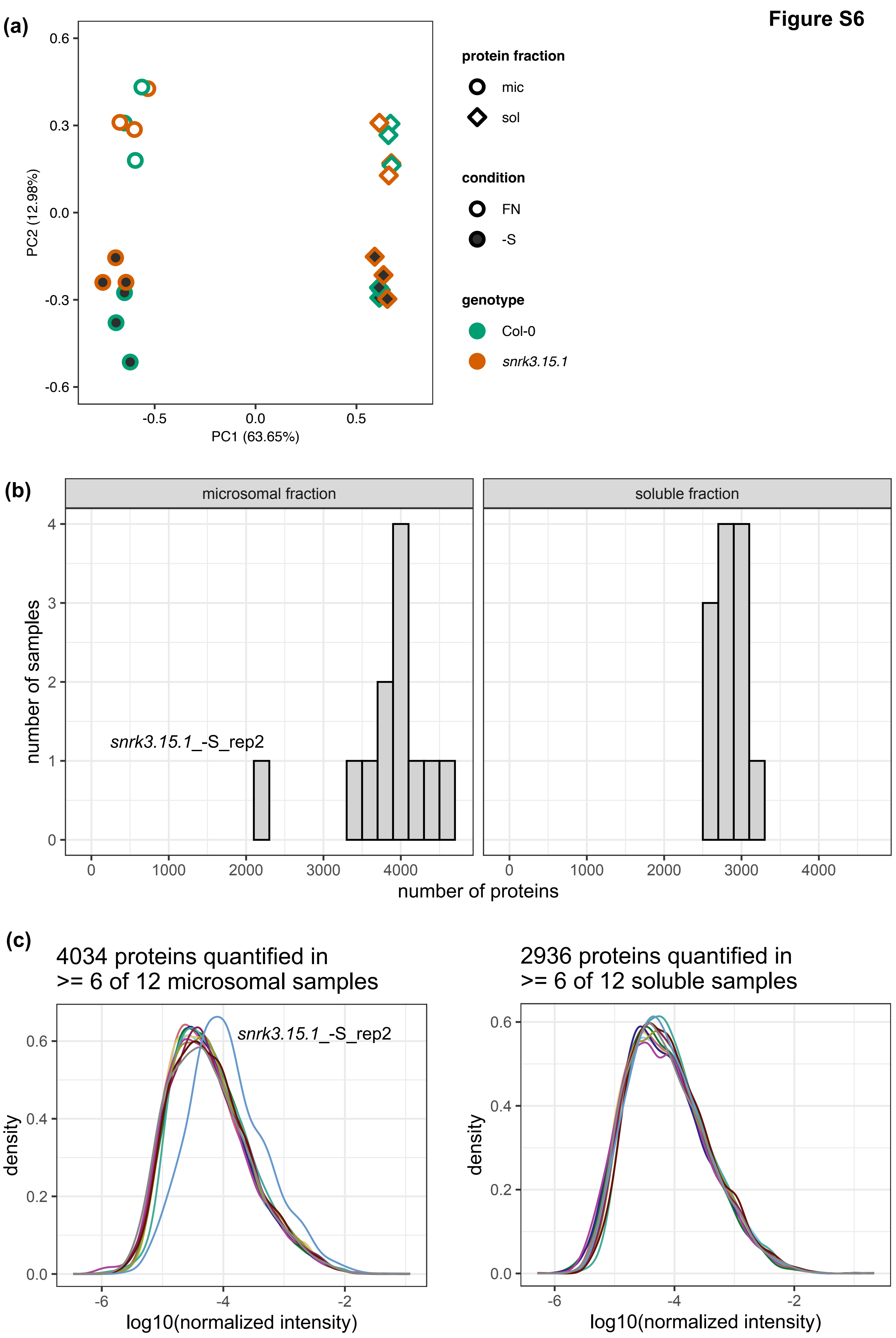
Figure S6
(a)
PC2 (12.98%)
PC1 (63.65%)
protein fraction
mic
sol
condition
FN
-S
genotype
Col-0
*snrk3.15.1*
(b)
microsomal fraction
soluble fraction
number of samples
number of proteins
*snrk3.15.1*_-S_rep2
(c)
4034 proteins quantified in
>= 6 of 12 microsomal samples
2936 proteins quantified in
>= 6 of 12 soluble samples
*snrk3.15.1*_-S_rep2
density
log10(normalized intensity)

**Figure S7**

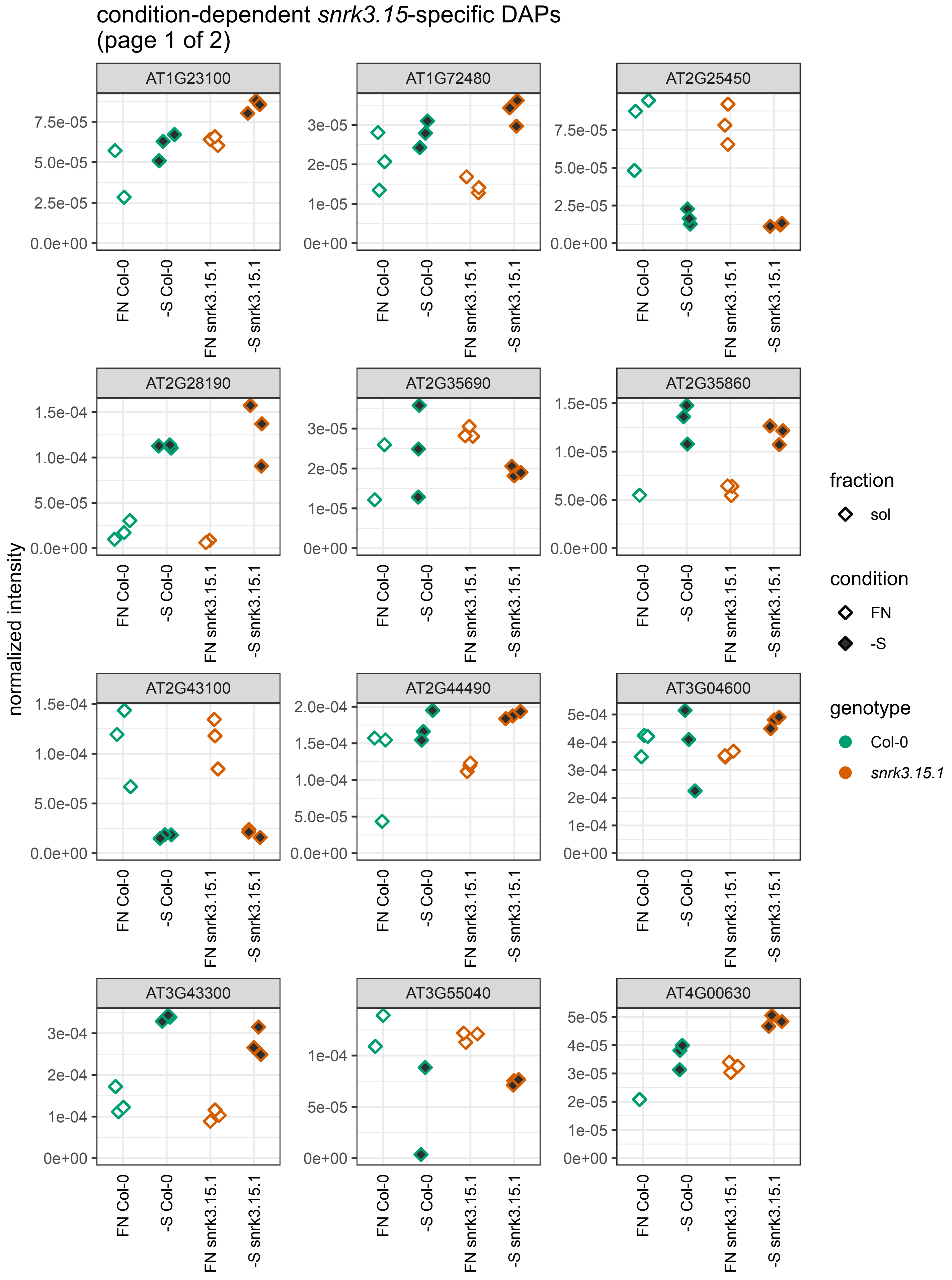

condition-dependent *snrk3.15*-specific DAPs
(page 1 of 2)
AT1G23100
AT1G72480
AT2G25450
AT2G28190
AT2G35690
AT2G35860
AT2G43100
AT2G44490
AT3G04600
AT3G43300
AT3G55040
AT4G00630
normalized intensity
FN Col-0
-S Col-0
FN snrk3.15.1
-S snrk3.15.1
fraction
sol
condition
FN
-S
genotype
Col-0
*snrk3.15.1*

condition-dependent *snrk3.15*-specific DAPs
(page 2 of 2)

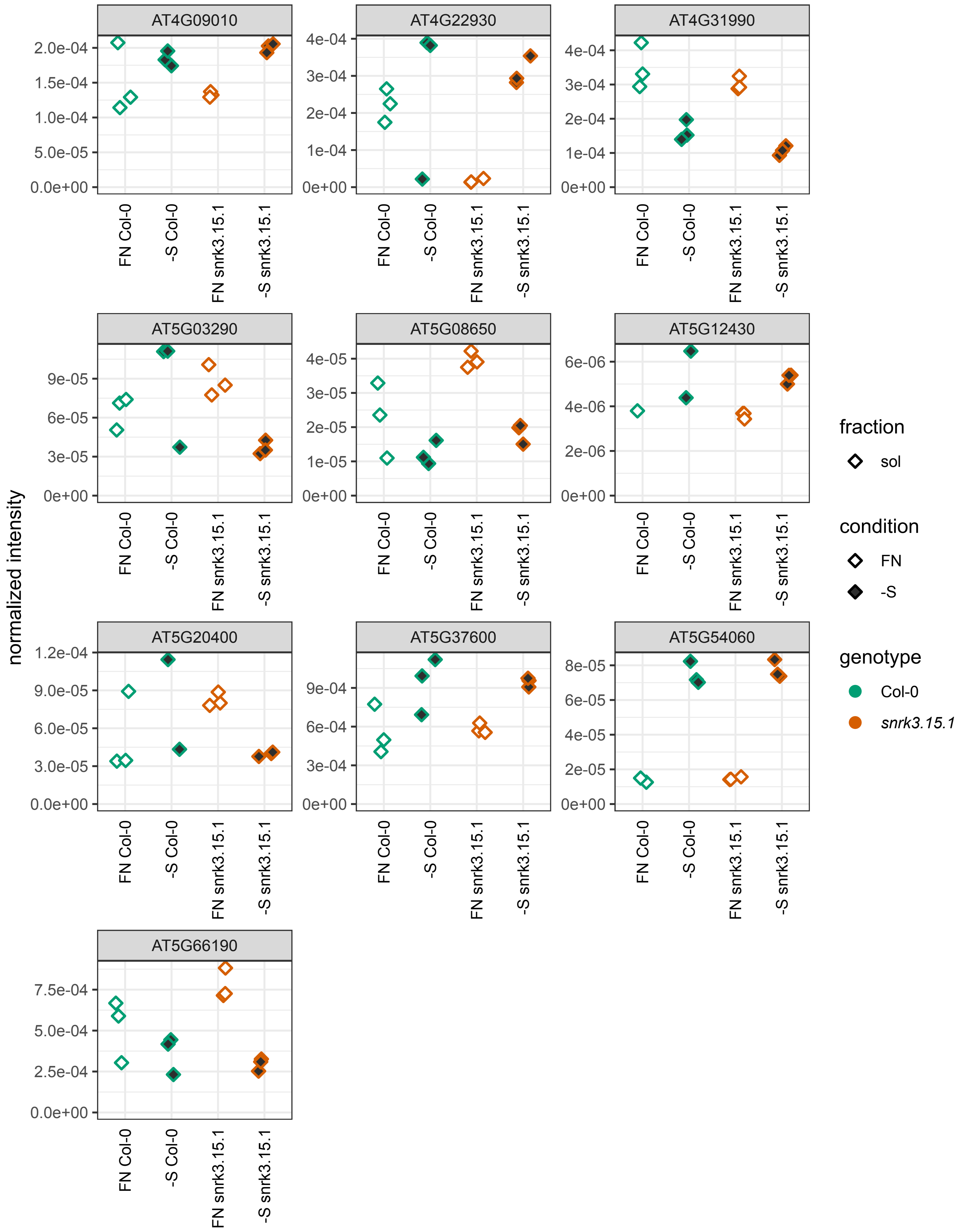

**Figure S8**

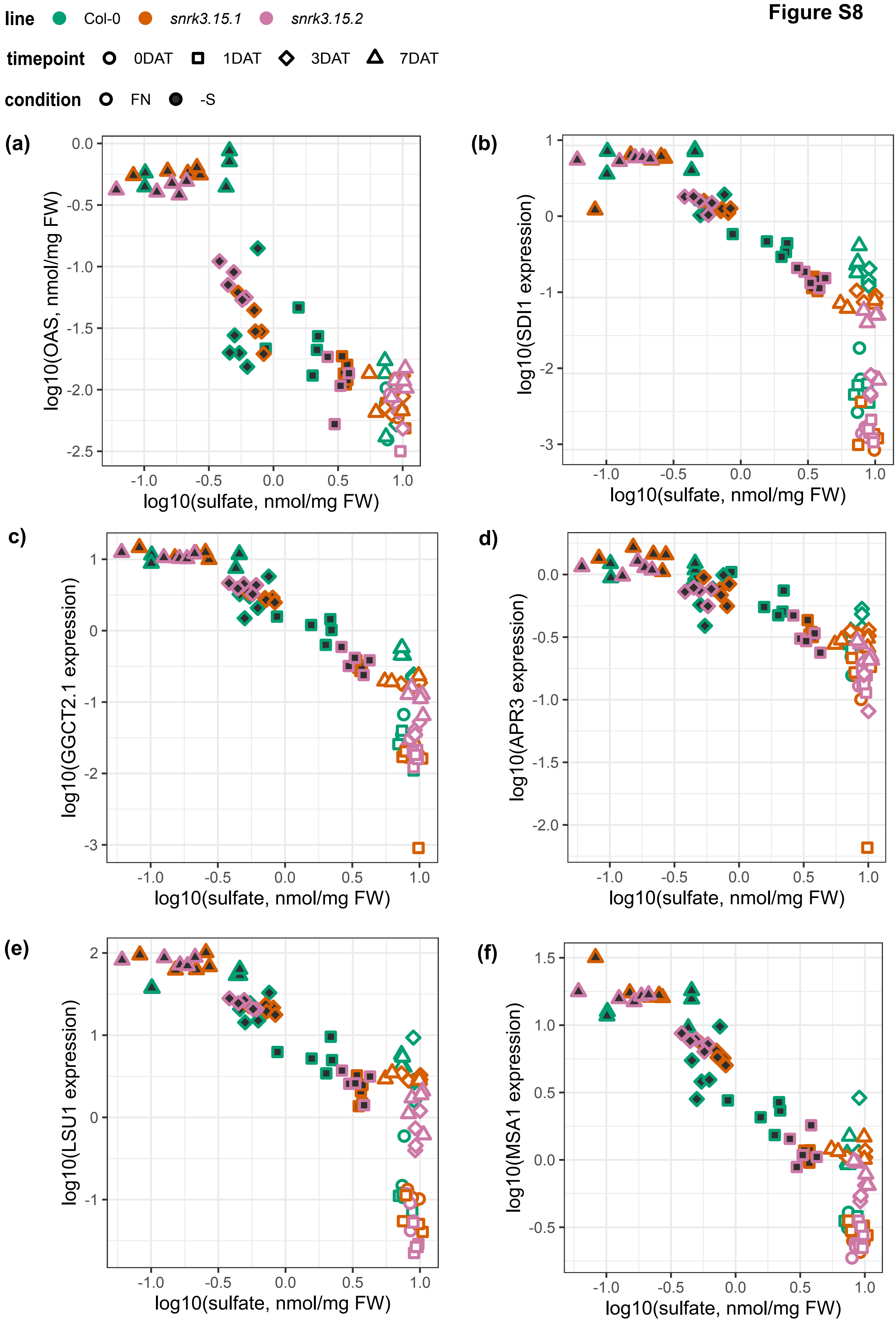
line Col-0 snrk3.15.1 snrk3.15.2
timepoint 0DAT 1DAT 3DAT 7DAT
condition FN -S
(a)
log10(OAS, nmol/mg FW)
log10(sulfate, nmol/mg FW)
(b)
log10(SDI1 expression)
log10(sulfate, nmol/mg FW)
c)
log10(GGCT2.1 expression)
log10(sulfate, nmol/mg FW)
d)
log10(APR3 expression)
log10(sulfate, nmol/mg FW)
(e)
log10(LSU1 expression)
log10(sulfate, nmol/mg FW)
(f)
log10(MSA1 expression)
log10(sulfate, nmol/mg FW)
