# Supplementary material for: SNRK3.15 Is a Crucial Component of the Sulfur Deprivation Response in Arabidopsis thaliana
Source: Plant Direct. 2025 Dec 28;10(1):e70132. doi: 10.1002/pld3.70132 (PMC12744938; doi:10.1002/pld3.70132)
Supplement: Supplementary file 4 — Table S2: Media composition. [file PLD3-10-e70132-s008.pdf]

Supplemental Table 2: Media Composition

Composition of modified MS medium. Macro- and micro-elements were added. MS agarose plates were made with either full sulfur supply (+S; 0.75 mM $MgSO_4$) or no sulfur supply (-S; 0.75 mM $MgCl_2$) using a low sulfur (≤ 0.15% sulfate) agarose (Biozym LE Agarose, Biozym Scientific GmbH, Hessisch Oldendorf, Germany).

**Macroelements**	**Final concentration**
$Ca(NO_3)2 \bullet 4H_2O$	1.5 mM
KNO_3	1 mM
KH_2PO_4	0.75 mM
$MgSO_4 \bullet 7H_2O$ (for FN)	0.75 mM
Fe-EDTA	1 mM
$MgCl_2 \bullet 6H_2O$ (for –S)	0.75 mM
**Microelements**	
$MnCl_2 \bullet 4H_2O$	10 µM
H_3BO_3	50 µM
$ZnCl_2$	1.75 µM
$CuCl_2$	0.5 µM
Na_2MoO_4	0.8 µM
KI	1 µM
$CoCl_2 \bullet 6H_2O$	0.1 µM
**Additives**	
MES hydrate	0.8 g/L
Low EEO agarose	8 g/L
Sucrose	10 g / L
pH 5.7 with KOH	
