# Supplementary material for: SNRK3.15 Is a Crucial Component of the Sulfur Deprivation Response in Arabidopsis thaliana
Source: Plant Direct. 2025 Dec 28;10(1):e70132. doi: 10.1002/pld3.70132 (PMC12744938; doi:10.1002/pld3.70132)
Supplement: Supplementary file 5 — Table S3: qRT‐PCR parameters. [file PLD3-10-e70132-s007.pdf]

Supplemental Table 3: qPCR parameters

Sample preparation for qRT-PCR. Reagent volume is written on the table below.

Reagent	Volume in RT-PCR reaction (5 µl)
cDNA sample (1:9)	0.5 µl
SYBR Green PCR Master Mix	2.5 µl
Primer mix (F + R) 1µM	2 µl

PCR program for qPCR.

	qPCR cycle	
	50 °C 2 min	
Initialization	95 °C 10 min	
Denaturation<br>Primer annealing and extension	95 °C 15 sec<br>60 °C 30 sec	40 times
	95 °C 15 sec	
Dissociation	60 °C 15 sec -> 95 °C 15 sec	
