# Supplementary material for: SNRK3.15 Is a Crucial Component of the Sulfur Deprivation Response in Arabidopsis thaliana
Source: Plant Direct. 2025 Dec 28;10(1):e70132. doi: 10.1002/pld3.70132 (PMC12744938; doi:10.1002/pld3.70132)
Supplement: Supplementary file 1 — Data S1: Peer Review. [file PLD3-10-e70132-s003.pdf]

SNRK3.15 is a crucial component of the sulfur deprivation response in Arabidopsis thaliana

Sarah J Whitcomb, Anastasia Apodiakou , Elmien Heyneke, Saleh Alseekh , Pinnapat Pinsorn, Sabine Metzger, Stanislav Kopriva, Waltraud Schulze, Rainer Hoefgen.

Decision Letter Round 1:

May 28, 2025

Dr. Sarah J Whitcomb
USDA-ARS Cereal Crops Research Unit
Madison, Wisconsin

RE: SNRK3.15 is a crucial component of the sulfur deprivation response in Arabidopsis thaliana

Dear Dr. Whitcomb:

Thank you for submitting to Plant Direct. All required reviews have been returned and we have now finished our evaluation of your manuscript. In light of the reviewers' and editor's comments, further revisions are needed before the paper can be accepted for publication in Plant Direct.

Please view the editors' and reviewers' comments below and use their suggestions as a guide while you work on your revision.

When uploading the revised version of this article, please be sure to include the following:

-A word document that contains your response to the reviewers. You should respond to each reviewer comment and note the changes made to the manuscript. If you do not agree with a reviewer's comment and choose not to make a suggested revision, please explain why. Please try to provide as complete an answer as possible to each reviewer's criticisms.
-A tracked changes document with each change highlighted
- A clean version of the latest version of the manuscript

To upload your revision, please click the link below.
https://plantdirect.msubmit.net/cgi-bin/main.plex?el=A5Lr1oj4A5pQa1I7A9ftdAPgDOxBlblOJuBqFzZ7QZ

In order to provide as timely a service as possible, we ask that your revision is resubmitted within three months after receipt of this request. If an extension is needed, please send a request, along with a brief explanation, to the editorial office at plantdirect@wiley.com .

Please note that, in addition to publishing reviewer comments, the author's responses to review comments will also be published alongside the final version of the paper. If you would not like the author's responses to be published, please contact the editorial office at plantdirect@wiley.com .

Thank you very much for giving us an opportunity to review your work. I look forward to receiving the next version.

Sincerely,
Marco Giovannetti
Gustavo MacIntosh
Editor, Plant Direct

-- Reviewer comments:
Reviewer #1:

Apodiakou and co-authors show that the regulatory roles of a serine/threonine protein kinase SNRK3.15 under sulfur deficiency using the knock-down mutant lines.

The authors compared wild-type Col-0 with two snrk3.15 knock-down mutants at normal and sulfur deficient nutrient conditions in diverse aspects: leaf and root phenotype, gene expressions, element concentrations, metabolite contents, proteome, and so on. Objective of each experiment in the manuscript is described enough to understand easily, and experiments employ well standardized methods with rational design. Data quality is sufficiently high. The manuscript sheds light on new kinase-dependent signaling pathways underlying sulfur deficiency response in plants, and thus is highly likely to satisfy the broad readership of Plant Direct.

Here are some comments:

1. Given leaf phenotypes and anthocyanin accumulations, it seems that up-regulation of SNRK3.15 under -S conditions at longer periods seems to be a negative event for plants coping with sulfur deficiency. The reviewer considered that SNRK3.15 is responsible for prompt response after sensing -S to boost sulfur assimilation and reallocation pathways, whereas prolonged S deficiency results in overboosting of these

pathways and thus complete depletion of sulfur sources comes earlier, then finally causes senescence. Is this understanding correct?

2. Several kinases such as cyclin-dependent kinase CDKA are involved in regulation of leaf cell size dependent on tissue growth or cell division cycle. The reviewer is interested in what majorly determines the larger rosette leaves in the snrk3.15 mutants under -S, cell size or number. It will easily be addressed by standard microscopic analysis.

3. The proteomic profiling provides a useful dataset confirming that changes in the protein levels of known low sulfur markers well correlates with transcriptome data in past studies. A limitation of the study is, however, that it was performed only at 3 days after transfer (DAT) to full nutrient or -S in the liquid shaking culture system. The snrk3.15 mutants show significant differences from Col-0 in the levels of sulfur-containing metabolites as well as sulfur deficiency marker gene expressions only at 1 DAT, while prolonged cultivation at -S induces larger rosette leaves and less accumulation of anthocyanins in the mutants. Extended proteomics studies at shorter or longer time periods would provide more relevant insights to the SNRK3.15 functions. Also, given SNRK3.15 belongs to the serine/threonine protein kinase family, specific profiling of phosphorylated proteins will be a better clue of identifying direct or indirect target proteins, as mentioned in Discussion (the reviewer understands phosphoproteomics is technically more difficult).

Reviewer #2:

The manuscript (MS) from Apodiakou et al. aims to investigate the role of serine/threonine protein kinase SNRK3.15 in the response to sulfate deprivation (-S) in Arabidopsis thaliana.
Previous studies have suggested a potential involvement of SNRK in the -S response, based on its transcriptional regulation under sulfur-deficient conditions. However, to date, no genetic evidence has supported this hypothesis.
Thus, in this study, the authors isolated two independent snrk3.15 knock-down mutant lines and demonstrated that seedlings with lower expression of SNRK3.15 gene are more tolerant to -S as confirmed in the phenotypical analyses.
To further support the involvement of SNRK3.15 in the response to -S, the authors evaluated several traits previously associated with sulfur deficiency. Overall, under -S conditions, the mutant lines showed higher chlorophyll content, along with the induction of several genes encoding for chlorophyll biosynthesis, while a marker gene for leaf senescence was downregulated compared to wild-type (WT) plants. Moreover, WT plants grown under -S showed an accumulation of anthocyanins at 7DAT (days after transfer) which was consistent with increased expression of genes involved in their

synthesis. However, mutant lines showed the opposite trend, suggesting that SNRK3.15 may be involved in some manner in their accumulation under -S. After one day of transfer to -S, WT plants drastically reduced sulfate content, while this reduction was attenuated in snrk3.15 mutants. Accordingly, sulfur deficiency marker genes were down-regulated in mutant lines. Finally, proteome analysis revealed a similar trend in mutant lines compared to WT plants, though the response was attenuated in snrk3.15 mutant lines.

Overall, the data presented indicate that the kinase SNRK1.3 participates in the -S signaling response in A. thaliana seedlings. The authors hypothesize that SNRK1.3 may be involved in an upstream regulatory mechanism, participating in the early response to sulfur deprivation.

This MS reveals some interesting results however, I found some concerns that need to be addressed before publication.

The MS would benefit if the authors could consider the following points:

Major issues:

1. My major concern regarding the MS is related to the statistical analyses performed in the study. I found confusing the presentation of the different comparisons between time/condition/genotype and requires clarification to ensure proper interpretation of the results. As indicated in the figure legends, letters indicate differences within each timepoint and condition. Could the authors clarify the post-hoc test was employed for mean comparisons? Moreover, authors state that T-tests were used to compare the means of each mutant line with the WT within each condition. Given this approach, I find more appropriate to use symbols rather than letters to denote significance, since mutant lines were not compared each other. I recommend the authors include all this information in Material and Methods section.

2. In line with the previous concern, the authors compared differential responses found in FN compared to -S; however, the statistical analyses presented do not compare both conditions. If I understood correctly, as indicated in the figure legends, letters a-c are used for FN, and letters d-f are used for -S. Thus, some conclusions are not supported by the data presented at the current form (e.g. lines 381-387; lines 404-425; lines 452-465; 506-516). Could the authors clarify this point?

3. Experimental data indicate that chlorophyll content in mutants under -S is 2-fold higher compared to WT plants (lines 384-386). Expression analyses of chlorophyll metabolism marker genes, lead the authors to conclude that SNRK3.15 is necessary for the strong transcriptional downregulation under -S of the chlorophyll biosynthesis genes CAO, CHLH, and GUN4 (Lines 404-425). According to the data presented, the total chlorophyll content of mutant lines and WT plants is not statistically significant at 3DAT; however the expression of CHLH and GUN4 genes showed an opposite trend. Could the authors discuss these results?

Minor issues:

1. I recommend that the authors avoid in-text references in the Results section. I find it more appropriate to include them in the Discussion section.
2. I recommend the authors to revise the statistical analysis. For instance, in figure 4, the expression of LSU1 gene at 3 DAT in snrk3.15. 2 is not statistical significant compared to WT. I recommend a thorough revision of the statistical analyses throughout the MS.
3. Figure S1 is missing.
4. Please correct the typo in gene nomenclature throughout the manuscript, as gene names should be italicized.
5. Revise Material and Methods section. Please correct the typos:
- Line 130. Substitute Lb13 by LBb 1.3.
- Line 135. FN denotes full nutrition. First time in text.
- Line 148. ddH2O
6. Revise in-text reference formatting (e.g., "Nikiforova et al." instead of V.J. Nikiforova).
7. To ensure the originality of the manuscript, I suggest rewrite some ideas in the introduction to further differentiate it from prior work of Aarabi et al. (2020).

Decision Letter Round 2:

October 4, 2025

Dr. Sarah J Whitcomb

USDA-ARS Cereal Crops Research Unit

Madison, Wisconsin

RE: SNRK3.15 is a crucial component of the sulfur deprivation response in Arabidopsis thaliana

Dear Dr. Whitcomb:

Thank you for submitting to Plant Direct. All required reviews have been returned and we have now finished our evaluation of your manuscript. In light of the reviewers' comments, further minor revisions are needed before the paper can be accepted for publication in Plant Direct.

Please view the reviewers' comments below and use their suggestions as a guide while you work on your revision.

When uploading the revised version of this article, please be sure to include the following:

-A word document that contains your response to the reviewers. You should respond to each reviewer comment and note the changes made to the manuscript. If you do not agree with a reviewer's comment and choose not to make a suggested revision, please explain why. Please try to provide as complete an answer as possible to each reviewer's criticisms.

-A tracked changes document with each change highlighted

- A clean version of the latest version of the manuscript

To upload your revision, please click the link below.

https://plantdirect.msubmit.net/cgi-bin/main.plex?el=A2Lr4oj7B7pQa1I4A9ftdAPgDOxBlblOJuBqFzZ7QZ

In order to provide as timely a service as possible, we ask that your revision is resubmitted within three months after receipt of this request. If an extension is needed, please send a request, along with a brief explanation, to the editorial office at plantdirect@wiley.com .

Please note that, in addition to publishing reviewer comments, the author's responses to review comments will also be published alongside the final version of the paper. If you would not like the author's responses to be published, please contact the editorial office at plantdirect@wiley.com .

Thank you very much for giving us an opportunity to review your work. I look forward to receiving the next version.

Sincerely,

Marco Giovannetti

Gustavo MacIntosh

Editor, Plant Direct

-- Reviewer comments:

Reviewer #1:

It was unfortunate that my concerns were not have hardly been addressed in the revised version.

2. The reviewer understands the difficulty during personnel movement and career transitions, these are not relevant to the article quality required for publication. Also, after revising statistical analysis, Fig. 1. exhibits no significant differences in the visible phenotypes between Col-0 and mutants. With only upregulation of SNRK3.15 and NIT3

expressions under -S, the section title 'SNRK3.15 has a role in S-deficiency response' would be overstatement.

3. The rationale of choosing 3DAT for proteomics should be stated somewhere in the main text in more detail, as described in the response letter. The reviewer understands the profiling of phosphorylated proteins can be a future challenge.

Reviewer #2:

The authors have adequately addressed all the points I raised in my previous review. The current version of the manuscript is significantly improved, with the statistical analysis and data presentation now being clear.

Revise some typos:

Line 37: FADH2. Please format the number as a subscript.

Line 239: Substitute "as described (Hubberten et al. 2012)" for "as described previously (Hubberten et al. 2012)".

Line 242: Substitute "described in (Almario et al., 2017) for "described in Almario et al. (2017).

Decision Letter Round 3:

December 4, 2025
Dr. Sarah J Whitcomb
USDA-ARS Cereal Crops Research Unit
Madison, Wisconsin

MSID: 2025-01796R2
MS TITLE: SNRK3.15 is a crucial component of the sulfur deprivation response in Arabidopsis thaliana

Dear Dr. Sarah Whitcomb:

I am pleased to inform you that your manuscript "SNRK3.15 is a crucial component of the sulfur deprivation response in Arabidopsis thaliana" has been accepted for publication in Plant Direct.

Your article will appear online in the next available issue of Plant Direct. To ensure your article gets published as quickly as possible, please pay attention to the steps detailed below. We have found that most of the delays happen at this stage, especially at the payment stage, so please respond as quickly as possible when prompted.

License Agreement: Once your article has been accepted it will move to Production and undergo admin and file checking - you may receive an email with any queries we have at this stage. When all required items are received by the publisher and queries resolved, the corresponding author will receive an email from Wiley's Author Services system which will ask them to log on at https://authorservices.wiley.com/bauthor and will present them with the appropriate license for completion. Your article cannot be published until both the signed license agreement and payment of the article fee have been received.

Payment of the Open Access Article Publication Fee: All articles published in Plant Direct are fully open access: immediately and freely available to read, download and share. Plant Direct charges a publication fee to cover the publication costs. The corresponding author for this manuscript should have already received a quote from the payments team (cs-openaccess@wiley.com) with the estimated article publication fee; please email cs-openaccess@wiley.com if this has not been received. The corresponding author should log on to the Wiley Author Services site, where the publication fee can be paid by credit card or an invoice. Pro Forma can also be requested. Payment of the publication charge must be received before the article will be published online.

Proofs: You will have the opportunity to look over your paper once more when you receive the author proofs for your article. The proofs will be with you in approximately two weeks. Please note that, in addition to publishing reviewer comments, the author's responses to review comments will also be published alongside the final version of the paper. If you would not like the author's responses to be published, please contact the editorial office at plantdirect@wiley.com .

Promotion of your article: You can help your research get the attention it deserves! Check out Wiley's free Promotion Guide for best-practice recommendations for promoting your work at www.wileyauthors.com/eeo/guide. And learn more about Wiley Editing Services which offers professional video, design, and writing services to create shareable video abstracts, infographics, conference posters, lay summaries, and research news stories for your research at www.wileyauthors.com/eeo/promotion.

Thank you again for your contribution to Plant Direct. If you have any questions, feel free to contact the editorial office at plantdirect@wiley.com .

Sincerely,

Marco Giovannetti
Gustavo MacIntosh
Editor, Plant Direct

Response Letter Round 1:

Dear Editors Giovannetti and MacIntosh,

We appreciate the constructive comments of the reviewers and the opportunity to improve our manuscript.

I think you will find that we have addressed the suggestions and critiques of both reviewers, and I hope you will find our responses and changes satisfactory for acceptance of our manuscript for publication in Plant Direct.

Sincerely,

Dr. Sarah J Whitcomb

USDA-ARS Cereal Crops Research Unit

Madison, Wisconsin, USA

Reviewer #1

1. Given leaf phenotypes and anthocyanin accumulations, it seems that up-regulation of

SNRK3.15 under -S conditions at longer periods seems to be a negative event for plants coping with sulfur deficiency. The reviewer considered that SNRK3.15 is responsible for prompt response after sensing -S to boost sulfur assimilation and reallocation pathways, whereas prolonged S deficiency results in overboosting of these pathways and thus complete depletion of sulfur sources comes earlier, then finally causes senescence. Is this understanding correct?

Our data support the conclusion that SNRK3.15 is important in Arabidopsis for both early adaptive responses to -S conditions and for later emergency salvage processes. Many of these responses can be characterized as “cooling down” of metabolism and growth, a shift to using other antioxidant compounds (like anthocyanins) than GSH, and eventually an initiation of senescence. It is plausible that it is adaptive for Arabidopsis to

slow down its growth and cool down its metabolism to survive a short period of Sdeficiency, and then to restart normal growth if S-homoestasis can be restored, and if not, to switch to a senescence and seed generation program. So while we agree with some points of the reviewer's summary in point 1, we would not necessarily characterize SNRK3.15 as having a negative impact on Arabidopsis plants coping with longer-term S-deficiency.

2. Several kinases such as cyclin-dependent kinase CDKA are involved in regulation of leaf cell size dependent on tissue growth or cell division cycle. The reviewer is interested in what majorly determines the larger rosette leaves in the snrk3.15 mutants under -S, cell size or number. It will easily be addressed by standard microscopic analysis.

The reviewer is interested in what determines the larger rosette area in the snrk3.15 mutants under -S, cell size or number. This is indeed an interesting question, and one that, as suggested by the reviewer, may be address by standard microscopic analysis. However, due to personnel movement and career transitions and lack of expertise with microscopic analysis, we chose not to pursue this suggested experimental expansion of our study. We agree that addressing the role of SNRK3.15 cell cycle/cell size regulation under -S would be an interesting follow-up study. We also chose to reduce the focus on potential size differences between Col-0 and snrk3.15 mutants because after changing our statistical approach (see point 3 below), the rosette area differences could no longer

be considered (statistically) significant.

3. The proteomic profiling provides a useful dataset confirming that changes in the protein levels of known low sulfur markers well correlates with transcriptome data in past studies. A limitation of the study is, however, that it was performed only at 3 days after transfer (DAT) to full nutrient or -S in the liquid shaking culture system. The snrk3.15 mutants show significant differences from Col-0 in the levels of sulfurcontaining metabolites as well as sulfur deficiency marker gene expressions only at 1

DAT, while prolonged cultivation at -S induces larger rosette leaves and less accumulation of anthocyanins in the mutants. Extended proteomics studies at shorter or longer time periods would provide more relevant insights to the SNRK3.15 functions. Also, given SNRK3.15 belongs to the serine/threonine protein kinase family, specific profiling of phosphorylated proteins will be a better clue of identifying direct or indirect target proteins, as mentioned in Discussion (the reviewer understands phosphoproteomics is technically more difficult).

We were only able to perform proteomic profiling on samples from a single timepoint after shifting seedlings to FN or -S media. 3 DAT was chosen for several reasons. First, we observed both differential early responses (at 1 DAT) and also differential later responses (at 7 DAT in the shaking culture system and at 11 DAT on plates). We reasoned that by choosing an intermediate timepoint we might observe meaningful outcomes of early responses to -S in the form of changes in protein abundance, and we might also observe differences in protein abundance that with sufficient time to act contribute to longer term -S responses. Second, although we observed numerous early responses to shifting seedlings to -S, we suspected that these responses were primarily due to fast-acting signaling cascades affecting enzyme and TF activity, rather than the primarily due to changes in their abundance. It is well established that transcript levels tend to respond to growth condition perturbations faster than protein levels, for example.

However, we agree that profiling -S and SNRK3.15 responsive proteins at additional timepoints would provide different and potentially interesting insights into SNRK3.15 function in the context of -S deficiency.

As the reviewer rightly pointed out and as we mentioned in the discussion (lines 691-694), SNRK3.15 belongs to the serine/threonine protein kinase family, and profiling of phosphorylated proteins may allow identification direct or indirect targets of SNRK3.15 phosphorylation. We actually performed phosphoproteomic profiling, however, phosphoproteomic profiling is technically more challenging than general proteomic

profiling, and the data quality was not sufficient for publication. With this publication we have demonstrated that SNRK3.15 is involved in both short-term and longer-term responses to -S conditions, and we hope that this will motivate others in the near future to search for direct and indirect kinase targets of SNRK3.15, especially under -S.

Reviewer #2

Major

1. My major concern regarding the MS is related to the statistical analyses performed in the study. I found confusing the presentation of the different comparisons between time/condition/genotype and requires clarification to ensure proper interpretation of the results. As indicated in the figure legends, letters indicate differences within each timepoint and condition. Could the authors clarify the post-hoc test was employed for mean comparisons? Moreover, authors state that T-tests were used to compare the means of each mutant line with the WT within each condition. Given this approach, I find more appropriate to use symbols rather than letters to denote significance, since mutant lines were not compared each other. I recommend the authors include all this information in Material and Methods section.

The reviewer stated that our description and presentation of the statistical analysis was unclear and needed to be improved. We greatly appreciate this criticism of our manuscript and the opportunity to improve it. We have remade Figures 1-5 and Supplemental Figures 1-3 to address the reviewer's comment. The data presented in these new figures are identical to the data presented in our original submission, but we modified our statistical approach. We have also updated the figure captions to properly describe the statistical approach presented in each figure.

In figure 1, we analyzed differences in means of sample groups corresponding to all combinations of two experimental variables: either timepoint x treatment (Figure 1a) or line x treatment (Figure 1c-f). Specifically, we performed ANOVA followed by post -hoc Tukey's pair-wise mean comparison testing, and presented the results with compact letter display, (alpha = 0.05).

In figures 2-5, data from experiments with 3 independent variables are shown: line x treatment x timepoint. Here differences in means between Col-0 and each snrk3.15 mutant were assessed by Welch two sample t-test. Comparisons between Col-0 and each snrk3.15 mutant were made within each treatment and timepoint. Asterisks are used to indicate significant differences based on the Welch test P-value.

2. In line with the previous concern, the authors compared differential responses found in FN compared to -S; however, the statistical analyses presented do not compare both conditions. If I understood correctly, as indicated in the figure legends, letters a-c are used for FN, and letters d-f are used for -S. Thus, some conclusions are not supported by the data presented at the current form (e.g. lines 381-387; lines 404-425; lines 452-465; 506-516). Could the authors clarify this point?

The reviewer pointed out that in the text we discuss differential responses to -S (comparing -S vs FN in Col-0 and in snrk3.15 mutants) but that we do not include this comparison in our statistical analysis. The reviewer specifically points to conclusions described in lines 381 – 387, lines 404 – 425, lines 452 – 465, and lines 506-516 in the original version of the manuscript.

Lines 378 – 382 (lines 381 – 387 in the original manuscript): In the current version of the manuscript, we removed the sentence, "For each of these proteins detected by LC/MS, the response to -S was determined" because it is protein abundance (at FN and -S) rather than protein abundance response (abundance at -S vs abundance at FN) that is visualized in Figure S4. We also added the word "qualitatively" to modify the differential responses to -S summarized in the following sentence. The lines in question now read, "none of the 31 proteins showed a qualitatively differential response to -S in snrk3.15 compared to Col-0 that was clearly explanatory of the differential chlorophyll content in these lines at 7 DAT (Figure S4)." We hope these changes sufficiently address the reviewer's concern because here we are not making a statistical assessment of whether a protein responds differently in snrk3.15 than in Col-0 to -S treatment and if anything we are at risk of understating our results, rather than overstating them.

Lines 393 – 415 (lines 404 – 425 in the original manuscript):

"response to -S" is specifically mentioned on line 394-5. Here we are comparing the very strong down regulation of CHLH and CAO transcripts in Col-0 under -S to the lack of a decrease in CHLH and CAO transcripts in snrk3.15.1 under -S. Looking at the data presented in Figure 2b,d, we do not consider this to be a contentious claim. The sentences in the manuscript read, "In Col-0, transcripts of CHLH, GUN4, and CAO are strongly downregulated as the duration of sulfate starvation increases (Figure 2b-d), and by 7 DAT, CHLH, GUN4, and CAO expression in Col-0 were 87%, 97%, and 77% lower than at FN, respectively. In contrast, CHLH and CAO transcript levels in snrk3.15 mutants were not decreased by the -S condition relative to FN at the same timepoints, a marked difference from their response to -S in Col-0 (Figure 2b,d)."

In lines 396-397 the difference in GUN4 expression between FN and -S is described. The sentence in the manuscript reads, "GUN4 expression was reduced by –S in snrk3.15 mutants at 7 DAT but to a lesser extent than in Col-0, resulting in 23-fold and 41-fold higher transcript levels than in Col-0 (Figure 2c)." Given the data presented in Figure 4c, we do not feel it is necessary to mention in the text the numerical, calculated fold change of GUN4 expression between -S and FN at 7 DAT in all 3 lines. Qualitatively, the fold change in Col-0 is clearly much greater than in snrk3.15.1 or in snrk3.15.2.

Lines 405-410 are structured similarly to lines 378-382, and we made equivalent adjustments to the text to address the error in language noticed by the reviewer. The sentences in the manuscript now read, "Eight proteins in the LC/MS dataset are annotated to the following gene ontology terms: chlorophyll catabolic process (GO:0015996), regulation of chlorophyll catabolic process (GO:0010271), negative regulation of chlorophyll biosynthetic process (GO:1902325). None of the eight proteins showed a qualitative differential response to -S (at 3 DAT) in Col-0 compared to snrk3.15 that could be clearly associated with lower chlorophyll content at 7 DAT (Figure S5).

Lines 440 – 447 (lines 452 – 465 in the original manuscript): We have modified the subheading associated with this section by removing “to -S”. The new subheading reads, “Changes to sulfur compound content and OAS-cluster gene expression at 1 DAT are attenuated in snrk3.15”. After careful re-reading of the lines of text referred to by the reviewer, we do not know how to further address the reviewer’s concern about this section.

Lines 484 – 495 (lines 506-516 in the original manuscript): Line 490 - 491 of the manuscript reads, “Notably, PC1 and PC2 do not clearly separate between Col-0 and snrk3.15.1 samples, suggesting that their global proteome responses to -S were similar.” This sentence references Figures S6a. It is our understanding that this kind of suggestion is appropriate from a PCA plot generally and is sufficiently supported by the data presented in Figure S6a. -S responses are also discussed in the remainder of the section pointed out by the reviewer (lines 493 – 495), but in this case, the data presented (Figure 6a) and described are explicitly response data (log2FC -S abundance vs FN abundance).

3. Experimental data indicate that chlorophyll content in mutants under -S is 2-fold higher compared to WT plants (lines 384-386). Expression analyses of chlorophyll metabolism marker genes, lead the authors to conclude that SNRK3.15 is necessary for the strong transcriptional downregulation under -S of the chlorophyll biosynthesis genes

CAO, CHLH, and GUN4 (Lines 404-425). According to the data presented, the total chlorophyll content of mutant lines and WT plants is not statistically significant at 3DAT; however the expression of CHLH and GUN4 genes showed an opposite trend. Could the authors discuss these results?

Differential biosynthetic enzyme transcript levels may logically precede differential compound levels because it may take time for changes in gene expression to be manifested (through multiple layers of regulatory control and biosynthetic steps) in compound levels. To make this more explicit, we have added text to the discussion,

lines 656 – 662 and 668-669.

Minor

1. I recommend that the authors avoid in-text references in the Results section. I find it more appropriate to include them in the Discussion section.

The reviewer recommended that we avoid in-text references in the results section. To comply with this suggestion, we removed references which were already cited in the introduction and/or the discussion sections, allowing for more focus on the results in the

results section. Citations which we consider useful to explain the rationale for certain experiments were retained in the result section text.

2. I recommend the authors to revise the statistical analysis. For instance, in figure 4, the expression of LSU1 gene at 3 DAT in snrk3.15. 2 is not statistical significant compared to WT. I recommend a thorough revision of the statistical analyses throughout the MS.

The reviewer recommended that the statistical analysis be revised. As described above (Reviewer #1, point 1), we reviewed and changed our statistical approach and its visualization. We greatly appreciate this criticism of our manuscript and the opportunity to improve it.

3. Figure S1 is missing.

Figure S1 was provided with our initial submission. However, thanks to the reviewer's comment, we noticed that panel 1a was missing from this supplemental figure in our initial submission. We have corrected the omission.

4. Please correct the typo in gene nomenclature throughout the manuscript, as gene names should be italicized.

We have checked the manuscript to confirm that gene names are italicized and made revisions where necessary. As a matter of convention, gene identifiers (e.g. AT5G01820) in Arabidopsis are typically not italicized. Two recent research articles published in Plant Direct follow this convention, https://doi.org/10.1002/pld3.70091,

https://doi.org/10.1002/pld3.70085. Therefore, we have left gene identifiers nonitalicized. Protein names, by convention are non-italicized, and we have followed that

convention as well.

5. Revise Material and Methods section. Please correct the typos:

- Line 130. Substitute Lb13 by LBb 1.3.

- Line 135. FN denotes full nutrition. First time in text.

- Line 148. ddH2O

The material and methods section has been reviewed and typos are corrected. Thank you for catching these typos.

6. Revise in-text reference formatting (e.g., "Nikiforova et al." instead of V.J. Nikiforova).

We reviewed the in-text references. V.J. Nikiforova et al. was changed to Nikiforova et al., W. Zhang was changed to Zhang et al., J Zhang was changed to Zhang et al. Thank you for catching these formatting errors.

7. To ensure the originality of the manuscript, I suggest rewrite some ideas in the introduction to further differentiate it from prior work of Aarabi et al. (2020).

We accept the reviewer's concern that the framing and or style of the introduction overlaps too closely with Aarabi et al. (2020). We have modified the parts of the introduction that we considered the most similar to those described in Aarabi et al. (2020).

Response Letter Round 2:

Dear Editors Giovannetti and MacIntosh,

Thank you for the opportunity to further improve our manuscript. While we still have differences of opinion with Reviewer #1, our revisions and rationale are detailed below. We hope you find these changes sufficient for acceptance of our manuscript for publication in Plant Direct.

Sincerely,

Dr. Sarah J Whitcomb

USDA-ARS Cereal Crops Research Unit

Madison, Wisconsin, USA

Reviewer #1

1. In their review of our initial manuscript, this reviewer expressed an interested in what determines the larger rosette area in the snrk3.15 mutants under -S, cell size or number. As we wrote in our previous response to reviewers, “this is indeed an interesting question, and one that, as suggested by the reviewer, may be address by standard microscopic analysis. However, due to personnel movement and career transitions and lack of expertise with microscopic analysis, we chose not to pursue this suggested experimental expansion of our study.” In the reviewer’s response to our revised manuscript, they state that “the reviewer understands the difficulty during personnel movement and career transitions, these are not relevant to the article quality required for publication.” In our assessment, addressing the role of SNRK3.15 cell cycle/cell size regulation under -S would be an interesting follow-up study, but it is not required for our manuscript to meet the standards for publication in Plant Direct.

2. [...] after revising statistical analysis, Fig. 1. exhibits no significant differences in the visible phenotypes between Col-0 and mutants. With only upregulation of SNRK3.15 and NIT3 expressions under -S, the section title 'SNRK3.15 has a role in S-deficiency response' would be overstatement.

The reviewer states there are no significant differences in the visible phenotypes between Col-0 and the snrk3.15.1 and snrk3.15.2 mutants, but we disagree. Lateral root density (a visual phenotype) responds strongly to -S treatment in Col-0 seedlings but not in snrk3.15 mutants (Figure 1d). The photo of representative seedlings under -S (Figure 1b) shows snrk3.15 mutants to be larger than Col-0. And under -S, the quantified rosette area of snrk3.15 mutant seedlings is also clearly larger than Col-0 seedlings (Figure 1c). [Line 344-345] “When grown on –S agar plates, the snrk3.15 mutants

displayed 1.7-fold greater total leaf area than Col-0”. In manuscript revision 1, the raw p-values

of the relevant comparisons were specified as being < 0.1. In the current revised version of the

manuscript, we have further specified [Lines 345-346] “(raw p-values = 0.047 and 0.056 for Col0 versus snrk3.15.1 and snrk3.15.2, respectively)”. As described in the Figure 1 caption,

“Differences among means were assessed by ANOVA followed by post hoc Tukey’s

pair-wise mean comparison testing with compact letter display, alpha = 0.05.” This

method of visualizing mean differences is conservative by design and incorporates pvalue adjustment for multiple comparisons. We consider a 1.7-fold difference in rosette

area between Col-0 and two independent snrk3.15 mutant lines to be a clear initial

indication of SNRK3.15 having a role in S-deficiency responses (even if the multiple

comparison adjusted p-values are not < 0.05), especially when considered together with

SNRK3.15 induction by -S conditions, the lack of lateral root density response to -S in

snrk3.15 mutants, and differential accumulation of NIT3 protein in response to -S.

3. The rationale of choosing 3DAT for proteomics should be stated somewhere in the

main text in more detail, as described in the response letter.

We thank the reviewer for this suggestion and have added our rationale to the results

section [Lines 485-488].

Reviewer #2

The typos have been corrected [Lines 36, 233, 236].
